# A Natural Alternative Treatment for Urinary Tract Infections: Itxasol©, the Importance of the Formulation

**DOI:** 10.3390/molecules26154564

**Published:** 2021-07-28

**Authors:** José M. Cela-López, Claudio J. Camacho Roldán, Gorka Gómez-Lizarraga, Vicente Martínez

**Affiliations:** Achucarro Basque Center for Neuroscience, Campus of Biscay, University of the Basque Country/Euskal Herriko Unibertsitatea, Parque Científico de la UPV/EHU, Edificio Sede, Barrio Sarriena, 48940 Leioa, Spain; Jose.cela@naturemimetix.com (J.M.C.-L.); claudio.camacho@naturemimetix.com (C.J.C.R.); gorka.gomez@naturemimetix.com (G.G.-L.)

**Keywords:** urinary tract infections, arbutin, umbelliferon, N-acetyl l-cysteine

## Abstract

Genito-urinary tract infections have a high incidence in the general population, being more prevalent among women than men. These diseases are usually treated with antibiotics, but very frequently, they are recurrent and lead to the creation of resistance and are associated with increased morbidity and mortality. For this reason, it is necessary to develop new compounds for their treatment. In this work, our objective is to review the characteristics of the compounds of a new formulation called Itxasol© that is prescribed as an adjuvant for the treatment of UTIs and composed of β-arbutin, umbelliferon and n-acetyl cysteine. This formulation, based on biomimetic principles, makes Itxasol© a broad-spectrum antibiotic with bactericidal, bacteriostatic and antifungal properties that is capable of destroying the biofilm and stopping its formation. It also acts as an anti-inflammatory agent, without the adverse effects associated with the recurrent use of antibiotics that leads to renal nephrotoxicity and other side effects. All these characteristics make Itxasol© an ideal candidate for the treatment of UTIs since it behaves like an antibiotic and with better characteristics than other adjuvants, such as D-mannose and cranberry extracts.

## 1. Introduction

Urinary tract infections (UTIs) affect any part of the urinary tract and may spread through the urinary tract towards the urethra, bladder and even the kidneys [1]. It has been estimated that around 150 million people suffer from UTIs annually [2], and they are associated with an increase in morbidity and mortality [3]. UTIs are more prevalent in women than in men, which is related to the short length of the urethra favoring bacteria colonization [4,5,6], and it has been estimated that 50–70% of women will suffer at least one urinary infection in their life [6]. Of women suffering from UTIs, 20–30% might suffer from a recurrent UTI, which is defined as recurrences of uncomplicated and/or complicated UTIs, with a frequency of at least three UTIs/year or two UTIs in the last six months [7]. UTIs in women are often associated with sexual intercourse [8], poor social conditions that limit access to female hygiene products for menstruation [9] and use of contraceptive devices such as diaphragms [10,11]. In the case of postmenopausal women, recurrent UTIs are related to the low levels of estrogens that produce changes in the vaginal microbiota [12]. In the case of men, UTIs are often related to prostatitis, prostate benign hyperplasia or any kind of urinary obstructive tract pathology [13,14]. In addition, UTIs are related to smoking, which is also associated with the development of bladder cancer, and it has been reported to increase the risk of suffering from UTIs by up to 50% [15].

The main bacteria isolated from cases of UTIs (80%) is *E. coli*, although other pathogens have been cultured, such as *Klebsiella pneumoniae* and *Pseudomonas aeruginosa* [16]. These infections represent a major number of hospital- and community-acquired infections, acute pyelonephritis being one of the major causes of hospitalization [6,17].

Causes of UTIs are related to sexual intercourse [18], and the incidence increases with age [19]. Other manifestations of UTIs are cystitis and pyelonephritis [20]. UTIs are often associated with the use of catheters [20,21], this being a major risk factor in hospital-acquired UTIs. UTIs are also related with radical cystectomy that is used for the treatment of different kinds of bladder cancer [22]. It has been estimated that around 35% of the patients after radical cystectomy surgery suffer from UTIs [23,24]. Of note, there are multiples cases of asymptomatic bacteriuria that do not require treatment, and they are not considered UTIs [6].

UTIs may be resolved spontaneously or treated with antibiotics [25]. Antibiotic resistance is a major problem nowadays [26]; in the European Union alone, it has been reported that annually, there are around 670,000 cases of infections related to multi-drug-resistant bacteria with 37,000 associated deaths, representing an enormous socioeconomic burden [27]. Regular use of antibiotics is related to nephrotoxicity due to interstitial nephritis, acute tubular necrosis and intratubular crystal deposition, which leads to an impaired kidney function [28]. Antibiotics also produce changes in the intestinal flora that trigger pathological processes, such as diarrhea, and alter immunity and metabolism; furthermore, gut bacteria have become a reservoir of genes for resistance to antibiotics [25].

As in other cases, UTI reinfections are recurrent, which leads to antibiotic resistance and, therefore, complications in treatment [29,30]. The recurrence of UTIs varies among different populations [16]. Children and young adult women usually suffer from having UTIs after a brief period following initial diagnosis [31,32].

Treatment of UTIs depends on various factors, such as the severity of the illness and the sex and age of the patient. The main first-line treatments are nitrofurantoin, fosfomycin trometamol, pivmecillinam and trimethoprim/sulfamethoxazole (TMP-SMX), with beta-lactams and fluoroquinolones being alternative therapies [33,34]. However, antibiotic usage produces nephrotoxicity in the case of gentamicin [35]. In addition, the occurrence of antibiotic resistance in UTIs is common [36,37], and this resistance is related to an increase in mortality due to microbial infections [38,39,40]. Moreover, regarding the use of antibiotics in patients with urothelial carcinoma, it has also been reported that patients under antibiotic treatment show a worse survival rate than that those who do not receive antibiotics [38].

One of the major problems related to the efficacy of antibiotics is the creation of biofilm by bacteria and fungus [41,42]. Biofilm promotes the propagation of these organisms and helps them to be more resistant to pharmacological treatments [43,44]. It has also been demonstrated that the formation of the biofilm plays a key role in catheter-associated infection [45]. This situation combined with the lack of new antibiotics requires effort to identify alternatives that might be used to combat recurrent infections. Regarding this concern, a new group of molecules called drug conjugates have been proposed as an alternative or a complement to the use of antibiotics alone [46]. These drug conjugates combine a sustained release, different kinds of antibiotics, and antibacterial activities as well as carrier composition [47].

It is important to underline that antibiotics come from natural sources and have been used to treat infections since ancient times. A milestone was reached with the discovery of penicillin by Sir Alexander Fleming in 1928 [48,49,50]. In this regard, Itxasol© is a new drug with a biomimetic origin that has recently been authorized by the Spanish Drugs Agency that belongs to Health Ministry (Agencia Española del Medicamento, authorization number C.N. 203621.5) to be used to treat UTIs as an adjuvant. Biomimetic might be defined as the science that studies nature as a source of inspiration for innovative technologies to solve human problems through models of systems (mechanics), processes (chemistry) or elements that imitate or are inspired by nature [51].

Itxasol© is composed of β-arbutin, umbelliferon (UMB) and N-acetyl l-cysteine (NAC). The three components of Itxasol© act as a natural antibiotic and potentially reduce inflammation, biofilm formation and the number of pathogenic microorganisms in the urinary tract.

Our main aim is to thoroughly review the knowledge regarding the three components of Itxasol©, a new drug compound, in relation to its efficacy to treat UTIs. Here, we analyze the mechanism of actions of the components of Itxasol©, namely, β−arbutin, UMB and NAC, and their main use related to the urinary tract.

## 2. Material and Methods

We searched PubMed, Web of Science (Clarative Analytics), and the Spanish databases Medes (https://medes.com/Public/Home.aspx, accessed on 1 June 2021) and IBECS (https://ibecs.isciii.es, accessed on 1 June 2021) for the following terms: umbelliferon/7-hydroxycoumarin and urinary tract/biofilm, arbutin and urinary tract/biofilm and *N*-acetylcysteine and urinary tract/biofilm (for the Spanish databases, the terms were written in Spanish). Table 1 summarizes the findings of this strategy search. Although single terms were found in Spanish databases, we did not find combinations of terms. After the original search, all abstracts were read, and those that were not related to the original search were discarded.

### 2.1. β-arbutin

**Figure 1:**
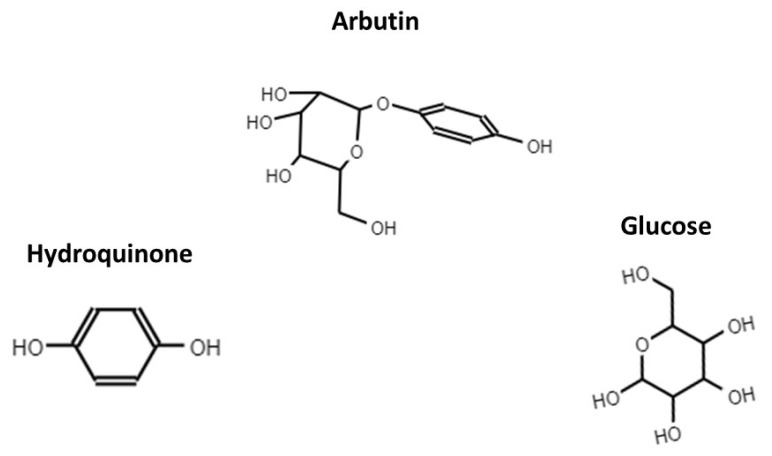
Chemical structure of arbutin, hydoquinone and D-Glucose.

Arbutin is a glycoside derived from extracts of leaves of *Arctostaphylos* uva-ursi, plants from genus *Bergenia* or other plants that belong to genus *Ainsliaea and Calluna*. These plants have been traditionally used for the treatment of urinary tract infections in Europe, America and Asiatic countries [52]. After its administration, arbutin is transformed into hydroquinone and glucose (Figure 1). The mechanism of action of hydroquinone (HQ) is related to the destruction of the bacteria wall, which leads to leakage of intracellular content and bacteria death [53]. One of the major advantages of β-arbutin treatment is that the amount of derived HQ that might reach the urinary tract after HQ excretion in urine is around 65% of the β-arbutin administrated [54].

The antibacterial activity of β-arbutin has been tested in several studies that demonstrated that it can destroy both Gram-negative and positive bacteria as well as fungi. In this list, we can find *Pseudomonas aeruginosa*, *Staphylococcus aureus*, *S*. *aureus* MRSA K31 (clinical, antibiotic-resistant strain), *Enterococcus faecalis*, *Escherichia coli*, *Escherichia coli* ESBL R194 (clinical, antibiotic-resistant strain), *Enterococcus faecalis* HLAR (clinical, antibiotic-resistant strain), *Bacillus subtilis* and *Candida albicans* [53,55,56]. In Table 2, we show the minimum inhibitory concentration of HQ against different pathogens.

In fact, in vivo, it has been reported in a randomized controlled trial that β-arbutin in combination with other plant extracts, such as berberine and birch, significantly reduced the incidence of recurrent cystitis [61]. The use of β-arbutin as a substitute for antibiotic treatment for UTIs is under study in a double-blind, randomized and controlled clinical trial on women aged 15 to 75 years old, but the results are yet to be published [62].

One concern regarding the safety of arbutin is that hydroquinone might be nephrotoxic [63,64]. Extract of strawberry tree *Arbutus unedo*, which contains arbutin, has been proven to be safe for the kidney in rats [65]. These extracts did not show any side effects in kidney function or affect the integrity of the DNA in the kidney cells. It has also been demonstrated that the administration of arbutin to humans was not toxic for lymphocytes, and it did not induce damage to the DNA of these cells [66].

Inflammation is an important consequence of UTIs. Regarding this effect, it has been demonstrated that β-arbutin reduces the kidney inflammation produced by the administration of lipopolysaccharide to rats. It was concluded that the anti-inflammatory effects of β-arbutin were mediated by inhibition of the Akt signaling pathway [67]. These results were dependent on the activity of arbutin, because when a specific arbutin activity inhibitor was administered, the beneficial effects of arbutin were abolished. Of note, arbutin, in a polyherbal mixture administered to diabetic rats, resulted in a decrease in blood sugar and level of cholesterol as well as cardiovascular risk, while it restored several histopathological changes produced by diabetes in the liver, pancreas and kidney [68]. The main actions of β-arbutin are summarized in Table 3.

### 2.2. Umbelliferon (UMB)

**Figure 2:**
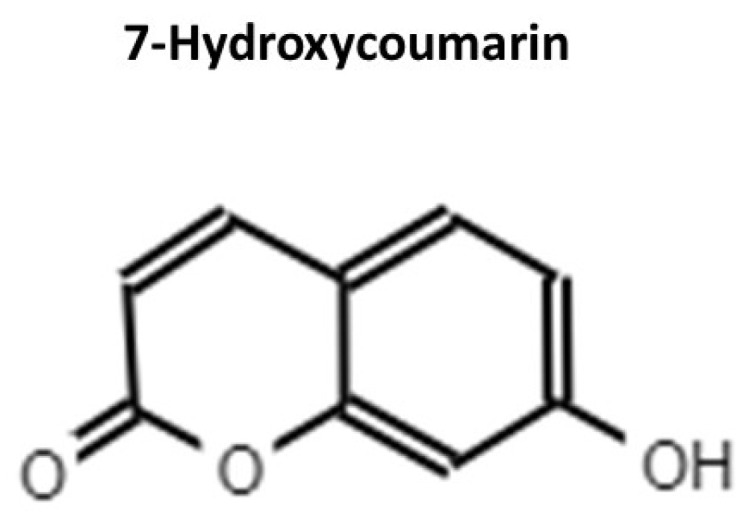
Chemical structure of UMB.

UMB (Figure 2) is a coumarin (7-hydroxycoumarin) that can be extracted from fruits [2] and plants and has demonstrated multiple activities, such as antitumoral, antioxidant (with the ability to quench free radicals), antihyperglycemic, anti-arthritic and anti-inflammatory activities, as well as hepatic and cerebral protective functions [17,69,70,71,72,73,74,75].

UMB activity as an antibiotic was described as earlier as 1978. However, it has not been used due to a lack of knowledge about its mechanism of action and its actions in the kidney [46]. Although knowledge of the antibiotic action of UMB is scarce, it has recently been demonstrated that derivates of UMB from plants of genus *Ferula* are useful against periodontal bacteria, inhibiting bacteria growth and biofilm formation [76]. In agreement with these data, it has been described that UMB can inhibit the formation of the biofilm created by *Staphylococcus* epidermidis, impairing intracellular adhesion by downregulation in the expressions of genes related to adherence function [77]. The MIC of UMB against different pathogens is shown in Table 4.

Coumarins are useful against fungus such as *C. albicans* [80,81]. Thus, this efficacy might lie in the fact that UMB can produce internal changes, such as an increase in ROS, DNA fragmentation and externalization of phosphatidyl serine, all of them being related to an increase in apoptosis [82]. The use of UMB to stop biofilm production in a model of *C. elegans* showed downregulation of the expression of genes related to the formation of filaments and adhesion as well as reducing the formation of biofilm in this nematode in general [83]. Besides fungi, it has also been reported that coumarins reduce the virulence and biofilm formation of *E. coli* O157:H7. Coumarins downregulate the expression of curli and motility genes, which results in fewer fimbriae and the production of biofilm [84]. In the same sense, UMB prevents the formation of biofilm by the methicillin-resistant Staphylococcus epidermidis strain at a dose of 500 mg/mL but without affecting the growth of this bacteria [77].

UMB has also been reported as a good nephron protector when it is used in combination with cisplatin, which is one of the chemotherapeutic agents used against different cancers but with limited use given its nephrotoxicity [85]. In a mouse model of cisplatin-induced acute kidney injury (AKI), it was demonstrated that UMB reduced the nephrotoxicity associated with cisplatin use. These effects were achieved by increased cell tubular proliferation upon enhanced expression of sox9 transcription factor. Another kidney-protective effect of UMB was the reduction of necrosis in kidney cells produced by the inhibition of the RIPK1/RIPK3/MLKL pathway. In a model of methotrexate-induced kidney injury, the administration of UMB reduced the expression of inflammation mediators P_38_MAPK and NF-κB and the pro-apoptotic molecules BAS and caspase3 while increasing anti-apoptotic levels of the BLC-2 molecule [86].

On the other hand, UMB has proven useful in treating the nephropathy produced in diabetic rat models induced with streptozotocin. In this model, UMB reduced the creatinine level in plasma, renal oxidative stress levels and levels of tissue and circulating TGF-β1 [1]. In another similar rat model study, in addition to the reduction of glucose blood levels, the beneficial effects of UMB were related to a decrease in inflammatory mediators, such as TNF-α, IL-6 and IL-1β; a reduction in the levels of mesenchymal–epithelial markers, such as podocin and CD2AP; and the reversal of some of the histopathological changes mediated by a diabetic status in the kidney [87]. In fact, this inhibition of the TGF-β1 pathway has also been reported in carbon tetrachloride-induced liver fibrosis in rats. After treatment with UMB, liver cells reduced the activation of the Smad/TGF-β1 pathway; downregulated the expression of NF-κB, collagen I and III and α-Smad; increased the levels of glutathione; and upregulated the expression of PPARγ. The histology result was a decrease in liver fibrosis [88]. The principal actions of UMB are summarized in Table 5.

### 2.3. N-Acetyl L-Cysteine (NAC)

**Figure 3:**
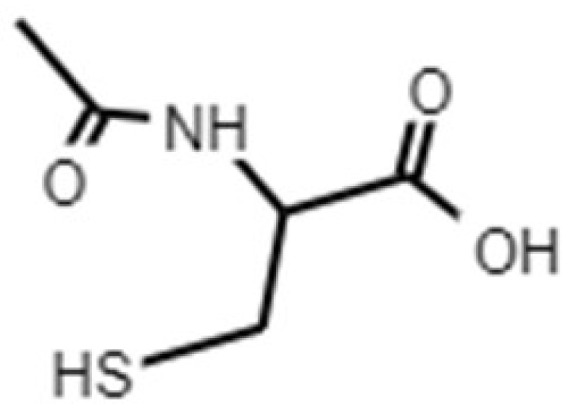
Chemical structure of NAC.

The NAC molecule (Figure 3) is well known for its antioxidant, anti-inflammatory and mucolytic actions [90,91]. Its mechanism of action is related to its ability to reduce the levels of reactive oxygen species (ROS), such as ^•^OH, NO_2_ and CO_3_**^−^**, given that it is a precursor of glutathione, a renowned natural ROS scavenger [92]. Moreover, there is compelling evidence regarding NAC as a molecule that destroys biofilms and also reduces their formation via bacteria and fungus [93,94]. Although the mechanism by which NAC reduces biofilm formation is still not clear, a recent report by Li and co-workers demonstrated that NAC can penetrate the bacteria wall, stopping protein synthesis and leading to bacteria death [95]. Table 6 summarizes the recent reports demonstrating the ability of NAC to both inhibit biofilm formation and destroy already formed ones. Besides being useful against UTIs, several pieces of evidence point out NAC as a kidney protector [96]. Moreover, a recent study demonstrated that the administration of NAC protected Wistar rats with a kidney injury against long warm renal ischemia. In particular, NAC administration resulted in an improvement in biochemical parameters and renal function when compared to the placebo group [97].

## 3. Discussion

UTIs have a high economic and social burden and affect large segments of the population, such as children, pregnant women, healthy pre- and post-menopausal women and patients undergoing catheterization and diabetics. In addition, there is a very high risk of relapse in UTIs, and it is common for these episodes to be caused by the same bacteria or something different [6]. Unfortunately, many of the current treatments fail to resolve UTIs and therefore lead to complications and relapses, and in many cases, patients also experience the adverse effects of medications [110,111].

**Figure 4:**
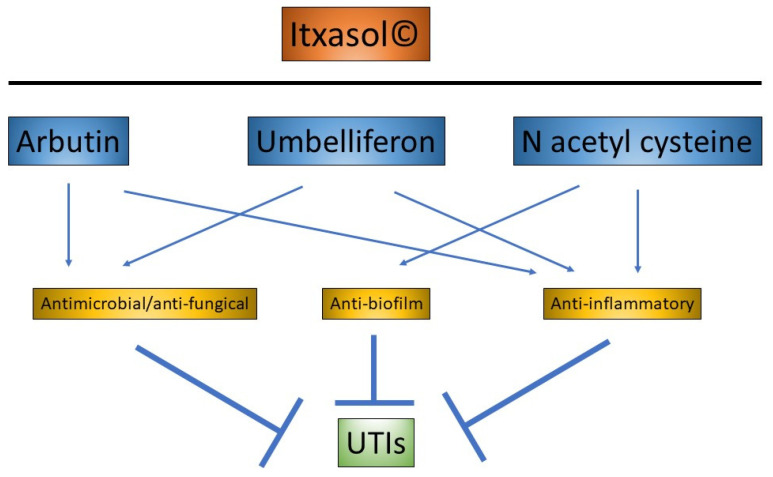
Combined mechanism of action of the three components of Itxasol©: β-arbutin, UMB and NAC.

Taken together, a new compound that combines antimicrobial and anti-inflammatory action and that does not have side effects is required to manage these diseases. Thus, in this study, we reviewed the main uses of arbutin, UMB and NAC, and the major actions of these three molecules are summarized in Figure 4.

There are new natural alternatives that, through an adequate formulation of different compounds, could contribute to solving this unmet need that constitutes the management of urinary tract infections. Itxasol© is a formulation that produces a synergistic effect thanks to its three components that act against urinary tract infections and improve diuresis. Itxasol© combines antibiotic properties of a natural bactericide and bacteriostat of the broad spectrum with antibiofilm (both prevention and elimination), nephroprotective, anti-inflammatory and antioxidant properties.

There are other coadjutants that have been used against UTIs, such as cranberry extracts and D-mannose or combinations of them or with extracts from other plants [111]. D-Mannose has been suggested as a coadjutant in the treatment of UTIs. However, this monosaccharide that acts by inhibiting the adherence of bacteria (*E. coli*) to urothelium [112] lacks antibacterial activity [113] and causes a considerable number of cases of diarrhea (around 8%) as a side effect [114]; moreover, its efficacy in regard to preventing or reducing UTI incidence remains clear [115]. UMB’s high bioavailability in the urinary tract is the characteristic that differentiates it from other coadjutants, such as curcumin (anthocyanosises), which can be found in red cranberry anthocyanosises. This feature makes it a suitable candidate for treating UTIs [116]. Of note, neither D-mannose nor cranberry extracts have an established dosage, and both are limited to the action of preventing the adherence of bacteria to the urothelium.

One of the limits of our review is that the combined effect of the molecules that form Itxasol© is not completely described. However, the antimicrobial activity of the three molecules has been tested in urinary catheters colonized with *Enterococcus faecalis*. In this study, it was demonstrated that these three molecules were able to reduce the formation of biofilms. In fact, after 72 h of treatment, the catheters treated with UMB (300 mg), arbutin (60 mg) and N-acetylcysteine (150 mg) showed a significant reduction in loaded biofilms and loaded bacteria [117]. It is important to highlight that the concentrations of the different compounds in the formulation are important since, in this study, UMB seems to work better at high doses (300 mg) accompanied by low doses of NAC (150 mg).

## 4. Conclusions

Itxasol© represents a promising tool in the treatment and prevention of ITUs, and it acts mainly as a broad-spectrum antibiotic protecting the kidney. It may be used for an extended time without generating bacterial resistance, which makes it an excellent alternative treatment to actual antibiotics and other coadjutants. The design of new controlled and randomized trials is necessary to confirm the potential of Itxasol© in comparison with current treatments.

## Figures and Tables

**Table 1:** Results of database search.

Term	PubMed	Web of Science
Umbelliferon and urinary tract	86	2
Umbelliferon and biofilm	9	1
7-Hydroxycoumarin and urinary tract	20	2
7-Hydroxycoumarin and biofilm	4	5
Arbutin and urinary tract	15	14
Arbutin and biofilm	2	3
*N*-acetylcysteine and urinary tract	807	54
*N*-acetylcysteine and biofilm	121	154

**Table 2:** Main MIC values for HQ.

Pathogen	MIC (μg/mL)	Gram	Reference
*Escherichia coli*	256	Negative	[57]
*Pseudomonas aeruginosa*	7.8	Negative	[58]
*Staphylococcus aureus*	15.6	Positive	[58]
*Staphylococcus aureus*	103	Positive	[59]
*Salmonella typhimurium*	512	Negative	[57]
*Bacillus cereus*	512	Positive	[57]
*Mycobacterium tuberculosis*	12.5	Positive	[60]

**Table 3:** Main actions of β-arbutin.

Action	Main Findings/Use	Reference
Antibacterial action	Inhibits *Pseudomonas aeruginosa* growth at 128 mg/mL	[56]
	Demonstration of antibacterial activity	[55]
	Arbutin destroys bacteria through wall cellular disruption (Gram + and Gram −)	[53]
	Reduction in bacterial load in prevention of UTI recurrence	[61]
	Clinical trial to reduce the use of antibiotics administering b-arbutin (results not yet published)	[62]
Anti-inflammatory	Attenuated damage induced by lipopolysaccharide in rat	[67]
Anti-diabetic	Ameliorates hyperglycemia, hyperlipidemia and histopathological changes in pancreas, kidney and liver in a diabetes rat model	[68]

**Table 4:** Main MIC values for UMB.

Pathogen	MIC (μg/mL)	Gram	Reference
*Escherichia coli*	1000	Negative	[78]
*Escherichia coli*	800	negative	[79]
*Shigella sonnei*	1000	Negative	[78]
*Salmonella typhimurium*	500	Negative	[78]
*Enterococcus faecalis*	1000	Positive	[78]
*Bacillus cereus*	62.5	Positive	[78]
*Staphylococcus aureus*	200	Positive	[79]

**Table 5:** Main actions of coumarins and their derivates.

Action	Main Findings/Use	References
Antifungicidal/antibiotic	Antifungicidal activity	[74,82,83]
	Decreases virulence and biofilm formation of *E.coli* O157:H7	[84]
	Impedes biofilm formation of methicillin-resistant *Staphylococcus epidermidis*	[77]
	Destroys periodontal bacteria and inhibits biofilm formation	[76]
	Antibiofilm properties (*Staphylococcus epidermidis)*	[77]
Antitumoral	Inhibits cell growth in lung carcinoma cell lines	[73]
	Induces cell cycle arrest in G0/G1 in human cell carcinoma	[75]
Anti-inflammatory	Reduction in inflammation in a model of brain damage in rats	[17]
Antihyperglycemic	Anti-diabetic effect in a diabetic mouse model induced with streptozotocin	[89]
Nephron protection	Reduction in the nephrotoxicity associated to cisplatin use	[85]
	UMB attenuates renal toxicity induced by gentamicin	[1,86]
	Enhances renal function in diabetic mouse model	[87]
Antifibrotic	Ameliorates the liver fibrosis signs induced by carbon tetrachloride (CCl4) in rats	[88]

**Table 6:** Recent reported studies on antibiofilm activities of NAC.

Organism	NAC Concentration	Reference
*C. albicans* *C. parapsilosis* *C. guilliermondii* *C. tropicalis* *C. glabrata*	10–50 mg/mL	[96]
*Pseudomonas* aeruginosa	0.15–0.23 mg/mL	[98]
*P. aeruginosa*	3–10 mg/mL	[99]
*P. aeruginosa*	32 mg/mL	[100]
*Candida albicans*	25 mg/mL	[101]
*Staphylococcus aureus*	0.5 mg/mL	[102]
*Stenotrophomonas* maltophilia	16–32 mg/mL	[103]
*Acinetobacter* baumannii	16–128 mg/mL	[104]
*Candida tropicalis*	1000 mg/mL	[105]
*Actinomyces naeslundii, Lactobacillus salivarius, Streptococcus mutans*, *Enterococcus faecalis*	25–100 mg/mL	[106]
*Pseudomonas aeruginosa*	10 mg/mL	[107]
*Staphylococcus pseudintermedius Pseudomonas aeruginosa Corynebacterium* spp. and β-hemolytic *Streptococcus* spp.	0.115–80 mg/mL	[108]
*Actinomyces naeslundii, Lactobacillus salivarius, Streptococcus mutans, Enterococcus faecalis*	0.78–1.56 mg/mL	[109]

## Data Availability

Data sharing not applicable.

## List of Abbreviations

UTIs	urinary tract infections
UMB	umbelliferon
NAC	N-acetyl L-cysteine
TMP-SMX	trimethoprim/sulfamethoxazole
MIC	minimum inhibitory concentration
